# Human Exposure to Electromagnetic Fields from Parallel Wireless Power Transfer Systems

**DOI:** 10.3390/ijerph14020157

**Published:** 2017-02-08

**Authors:** Feng Wen, Xueliang Huang

**Affiliations:** School of Electrical Engineering, Southeast University, No. 2 Sipailou, Nanjing 210096, China; wf133@163.com (F.W.); xlhuang@seu.edu.cn (X.H.); Tel.: +86-25-8379-3705 (F.W.); +86-25-8379-2260 (X.H.)

**Keywords:** wireless power transfer (WPT), human tissues, induced electric field, specific absorption rate (SAR)

## Abstract

The scenario of multiple wireless power transfer (WPT) systems working closely, synchronously or asynchronously with phase difference often occurs in power supply for household appliances and electric vehicles in parking lots. Magnetic field leakage from the WPT systems is also varied due to unpredictable asynchronous working conditions. In this study, the magnetic field leakage from parallel WPT systems working with phase difference is predicted, and the induced electric field and specific absorption rate (SAR) in a human body standing in the vicinity are also evaluated. Computational results are compared with the restrictions prescribed in the regulations established to limit human exposure to time-varying electromagnetic fields (EMFs). The results show that the middle region between the two WPT coils is safer for the two WPT systems working in-phase, and the peripheral regions are safer around the WPT systems working anti-phase. Thin metallic plates larger than the WPT coils can shield the magnetic field leakage well, while smaller ones may worsen the situation. The orientation of the human body will influence the maximum magnitude of induced electric field and its distribution within the human body. The induced electric field centralizes in the trunk, groin, and genitals with only one exception: when the human body is standing right at the middle of the two WPT coils working in-phase, the induced electric field focuses on lower limbs. The SAR value in the lungs always seems to be greater than in other organs, while the value in the liver is minimal. Human exposure to EMFs meets the guidelines of the International Committee on Non-Ionizing Radiation Protection (ICNIRP), specifically reference levels with respect to magnetic field and basic restrictions on induced electric fields and SAR, as the charging power is lower than 3.1 kW and 55.5 kW, respectively. These results are positive with respect to the safe applications of parallel WPT systems working simultaneously.

## 1. Introduction

Wireless power transfer (WPT) technology using magnetic field coupling and offering wireless mid-range power transmission has been used for a variety of applications such as implantable biomedical devices, mobile electronics, household appliances, and electric vehicles [1,2,3,4]. However, as WPT technology is becoming more widely used in high power applications, human exposure to time-varying electromagnetic fields (EMFs) accordingly increases. As a result, internal electric fields induce body currents in tissues so that the nervous system can be stimulated depending on the frequency involved. In addition, the electromagnetic field leaked from the WPT systems may cause the false operation of other electronic devices, so it is necessary to quantitatively evaluate the electromagnetic interference. The leakage of electromagnetic field from WPT systems should also comply with the related regulations to make the WPT technology safe and accessible. Studies have been carried out by researchers in different scenarios such as housing environments [5,6], electrical vehicles [7,8,9,10,11,12], and bio-implanted applications [13,14,15,16,17,18,19,20]. Human models of adults, children, and pregnant woman [21,22,23,24,25,26,27,28,29] have also been built to estimate possible biological threats.

In previous studies, human exposure in a single wireless power transfer (WPT) scenario is numerically examined with respect to the induced electric field and specific absorption rate to determine the optimal operating frequency and maximum obtainable power without exceeding the exposure limits. In addition, measurement, numerical methods, and coil design have also been assessed in prior studies. However, there exists great possibilities with respect to power supply using multiple WPT systems working simultaneously and closely for household appliances and electric vehicles in parking lots. These WPT systems may work synchronously or asynchronously with phase difference. The magnetic field leakage from the WPT systems is also varied due to unpredictable asynchronous working conditions, which can induce an electric field in human bodies standing around the WPT systems and potentially pose adverse effects to health. In this study, the magnetic field leakage from parallel WPT systems working with phase difference is predicted by combining the circuit and three-dimensional (3D) finite element analysis (FEA) models. The induced electric field and the specific absorption rate (SAR) in a human body standing in the vicinity are also evaluated. Computational results are compared with the limit prescribed in the regulations established to limit human exposure to time-varying electromagnetic fields (EMFs) such as the International Committee on Non-Ionizing Radiation Protection (ICNIRP) guidelines, and regulations of the Institute of Electrical and Electronic Engineers (IEEE) International Committee on Electromagnetic Safety. By summarizing the results, the distribution regularities of the magnetic field leakage, induced electric field and SAR in the human body are achieved. This study can contribute to safe use of parallel WPT systems working simultaneously.

## 2. Computational Models and Methods

### 2.1. FEA Model and Cricuit Model

**Figure 1:**
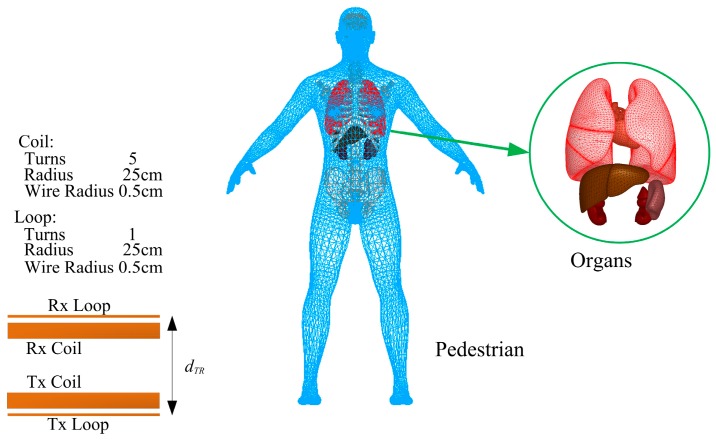
Three-dimensional (3D) finite element analysis (FEA) numeric model of adult and wireless power transfer (WPT) coils. Rx: pick-up; Tx: transmission.

Figure 1 depicts the 3D FEA numeric model of adult and WPT coils. The precise human anatomic finite element model (male, 30 years, 180 cm, 70 kg), has a resolution of 2 mm and consists of more than 30 types of tissues such as brain, heart, liver, kidney and so forth. The tissue dielectric properties are obtained from the IT’IS tissue database [30] which is primarily based on the work of Gabriel [31,32,33]. The four WPT coils are 25 cm in radius and are wound with copper wire with a radius of 0.5 cm. The number of turns in the driving coil (Tx loop) and pick-up coil (Rx loop) is 1 and in the transmission coil (Tx coil) and receiving coil (Rx coil) this value is 5. The distance between the loop and the coil for both Tx and Rx is 1 cm, the distance between the Tx and Rx coil (*d_TR_*) is 30 cm.

**Figure 2:**
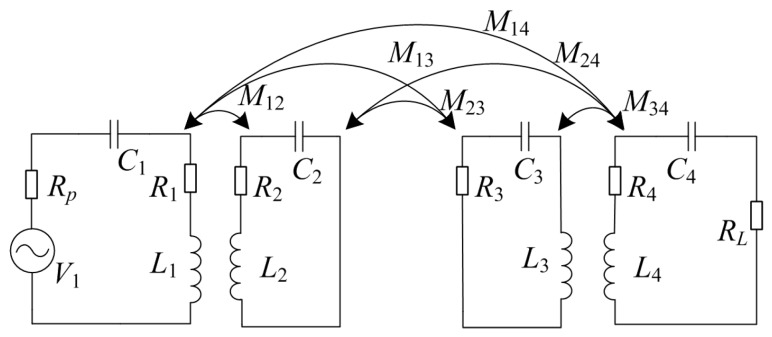
Equivalent circuit model of the WPT system.

Figure 2 shows the equivalent circuit model of the WPT system based on mutual inductance theory. There, *L*_1_, *L*_2_, *L*_3_, and *L*_4_ are the self-inductance of the Tx loop, Tx coil, Rx coil, and Rx loop, respectively, *C*_1_, *C*_2_, *C*_3_, and *C*_4_ are the respective resonance capacitance of the coils, *R*_1_, *R*_2_, *R*_3_, and *R*_4_ the equivalent resistances of the coils, *R_p_* is the internal resistance of the power supply (50 Ω), *R_L_* the load resistance (10 Ω), and *V*_1_ is the excitation voltage source. *M_mn_* is the mutual inductance of any pair of coils, with *m*, *n* ϵ {1, 2, 3, 4}, *m* ≠ *n*.

### 2.2. Computational Methods

According to the Kirchhoff’s law, the impedance matrix of the system can be expressed as:

(1) $$\left[\begin{array}{cccc} Z_{11} & Z_{12} & Z_{13} & Z_{14}\\ Z_{12} & Z_{22} & Z_{23} & Z_{24}\\ Z_{13} & Z_{23} & Z_{33} & Z_{34}\\ \;Z_{14} & Z_{24} & Z_{34} & Z_{44} \end{array}\right]\cdot \left[\begin{array}{c} I_{1}\\ I_{2}\\ I_{3}\\ I_{4} \end{array}\right]=\left[\begin{array}{c} V_{1}\\ 0\\ 0\\ 0 \end{array}\right]$$

where *Z_nn_* = *R_nn_* + *jX_nn_*, *Z_mn_* = *jX_mn_*, *X_mn_* = *ωM_mn_*, *m*, *n* ϵ {1, 2, 3, 4}, *m*≠ *n*, *X_nn_* = *ωL_n_* – 1/(*ωC_n_*), *n* ϵ {1, 2, 3, 4}, *R*_11_ = *R_p_* + *R*_1_, *R*_22_ = *R*_2_, *R*_33_ = *R*_3_, *R*_44_ = *R_L_* + *R*_4_, *V*_1_ is voltage of the excitation source, and *I*_1_, *I*_2_, *I*_3_, and *I*_4_ are currents in the coils which can be calculated using Equation (2):

(2) $$\begin{array}{c} I_{1}=I_{4}(Z_{44}Z_{23}{}^{2}+Z_{22}Z_{34}{}^{2}-Z_{22}Z_{33}Z_{44})/Z_{12}Z_{23}Z_{34}\\ I_{2}=I_{4}Z_{12}(Z_{33}Z_{44}-Z_{34}{}^{2})/(Z_{23}Z_{34})\\ I_{3}=-I_{4}Z_{44}/Z_{34}\\ I_{4}=V_{1}Z_{12}Z_{23}Z_{34}/(-Z_{12}{}^{2}Z_{34}{}^{2}+Z_{33}Z_{44}Z_{12}{}^{2}+Z_{11}Z_{44}Z_{23}{}^{2}+Z_{11}Z_{22}Z_{34}{}^{2}-Z_{11}Z_{22}Z_{33}Z_{44}) \end{array}$$

The transmission power of the WPT system provided by the Rx loop can be written as:

(3) $$P_{o}={\left|I_{4}\right|}^{2}\cdot R_{L}$$

The specific absorption rate (SAR) can be obtained using Equation (4):

(4) $$SAR=0.5\cdot \sigma_{t}{\left|E_{\max}\right|}^{2}/\rho_{t}$$

where σ*_t_* and *ρ_t_* are the conductivity and the mass density of the human biological tissue, respectively, and *E*_max_ represents the magnitudes of the complex electric field amplitudes in tissue.

In this paper, the FEA method is used to investigate the induced current density/in-situ electric field in the human body standing in the exposure scenario. During mesh generation in the FEA tool ANSYS HFSS (which utilizes the full-wave finite element method, FEM), a continuous geometry domain is discretized into small three-dimensional tetrahedral and two-dimensional triangular elements. The method has been proven to be effective by previous studies performed by other researchers and our group [34]. Using HFSS, the key parameters of coils in Figure 1 are calculated and shown in Table 1. The coils are matched at 1 MHz by connecting series capacitors. The coupling coefficients of the Tx loop and coil, Tx and Rx coil, and Rx loop and coil are denoted as *k*_12_, *k*_23_, and *k*_34_, respectively. Table 2 summarizes parameters of some typical tissues at the operating frequency of the system, which are taken from the IT’IS tissue database.

## 3. Exposure Scenarios

**Figure 3:**
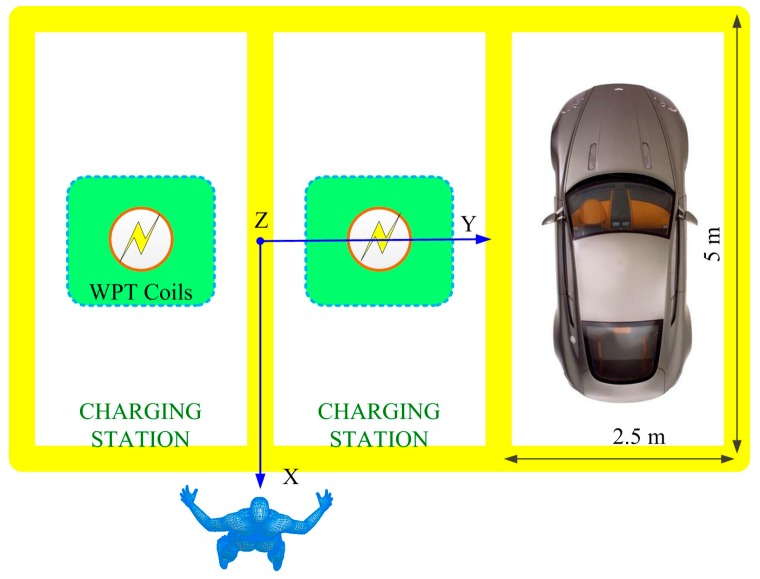
WPT systems for electric vehicle charging.

Scenarios of multiple WPT systems working simultaneously are considered in this study. These situations often occur in power supply for household appliances and electric vehicles in parking lots. We chose two parallel WPT systems for electric vehicle charging as shown, for example, in Figure 3. Although the power frequency is not in the common band established by SAE TIR J2954 [35], the research is still significant for general cases. The magnetic field leakage, induced electric field and SAR in the human body are predicted under different exposure scenarios.

### 3.1. Magnetic Field Leakage

Magnetic field level is calculated at 1 m above the ground. Reference levels for general public exposure to time-varying magnetic fields at 1 MHz are 0.73 A/m by the ICNIRP 1998 [36], 16.3 A/m by the IEEE [37], and 21 A/m by the ICNIRP 2010 [38]. We choose the most stringent restriction by ICNIRP 1998 to calculate the safe zone as the charging power equals 3.3 kW. The root mean square (RMS) value of voltage source *V*_1_ equals 925.5 V, and the currents in WPT coils listed in Table 3 are calculated using Equations (2) and (3).

As with the two WPT systems working out of sync with a phase difference of Δα in power voltage, currents in loops and coils of the two systems are also different in phase, which causes a Δα phase difference of the magnetic flux density produced by the two systems. The magnetic field that is generated by the currents of the two WPT systems can be expressed using Equation (5),

(5) $$\begin{array}{c} B_{1}=B_{1m}\cos(\omega t)\\ B_{2}=B_{2m}\cos(\omega t+\alpha )\\ B_{t}=\left|{\vec{B}}_{t}\right|=\left|{\vec{B}}_{1}+{\vec{B}}_{2}\right| \end{array}$$

**Figure 4:**
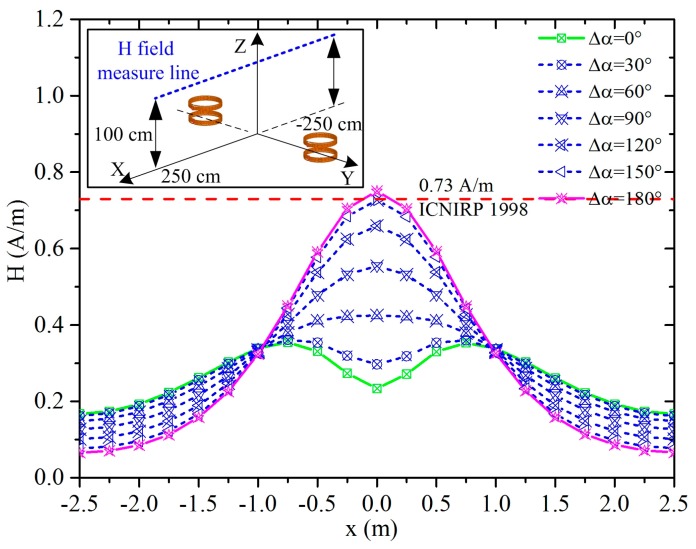
Magnetic field intensity (A/m) of two parallel WPT systems with phase difference along the measure line. ICNIRP: International Committee on Non-Ionizing Radiation Protection.

**Figure 5:**
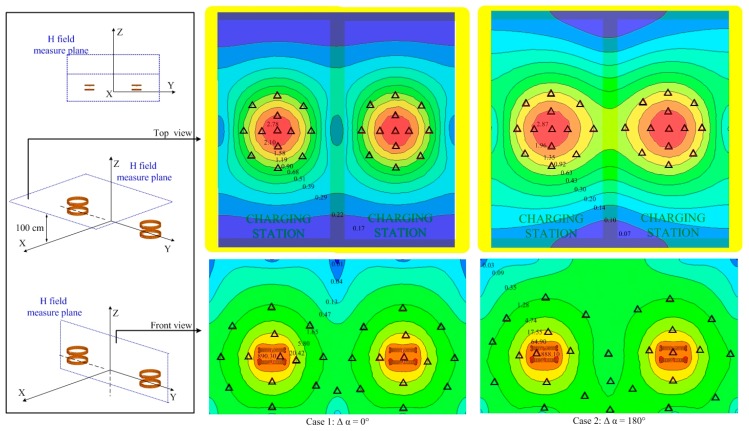
Magnetic field intensity (A/m) of two parallel WPT systems on the measure plane.

The magnetic field intensity along the measure line is shown in the illustration in Figure 4 using the two WPT systems with a phase difference of 30°, 60°, 90°, 120°, 150° and 180°. As for the two WPT systems working in-phase, the field intensity in the middle region between the two WPT coils is weaker, and it is stronger in the peripheral regions around the WPT coils. As Δα equals 180°, the peak value of the magnetic field intensity equals 0.75 A/m, which exceeds the reference level. The distributions of the magnetic field intensity on the XY plane as z = 1 m and on the YZ plane as x = 0 are shown in Figure 5, the regions where the field intensity exceeds the reference level are marked by triangles. In the fore and aft regions around the vehicles, the field intensity is relatively lower, while the values are closer to the limit in other areas such as vehicle side doors which drivers or passengers must access. By comparing the magnetic field distributions of the two cases in Figure 5, we can conclude that the middle region between the two WPT coils is safer for case 1 as the two WPT systems are working in-phase, and the peripheral regions are safer for case 2 as the systems are working anti-phase.

**Figure 6:**
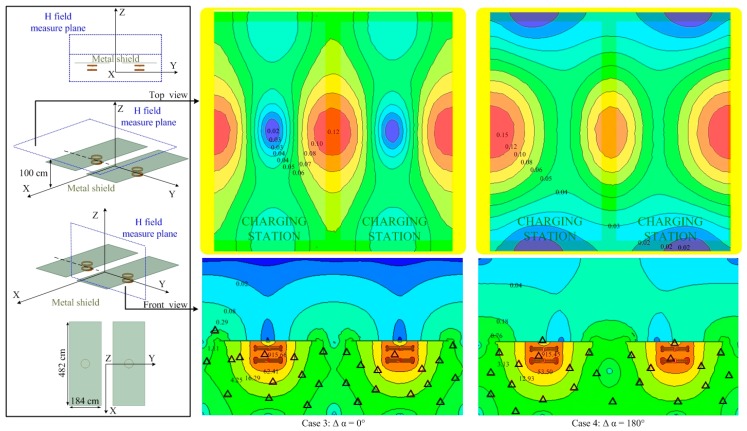
Magnetic field intensity (A/m) of two parallel WPT systems with a metal shield.

**Figure 7:**
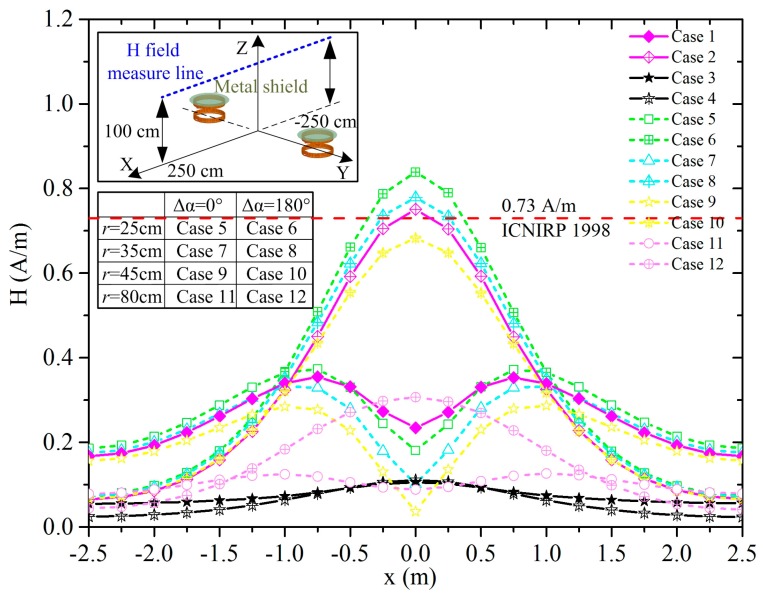
Magnetic field intensity (A/m) of two parallel WPT systems with a metal shield along the measure line in illustration.

In the scenario of wireless charging for electric vehicles, metal plates are generally considered for the bottom and body surface of the vehicle, providing an excellent shield effect for leakage of the magnetic field. We have taken the aluminum plates right above the WPT coils as the illustration shows in Figure 6. The two WPT systems are fine-tuned respectively to maintain a stable charging power of 3.3 kW. The magnetic field intensity shown in Figure 6 indicates that metal plates of large size will indeed shield the magnetic field leakage well; it is safe around the vehicle in both cases 3 and 4. However, in common applications, it will be difficult to acquire shielding from metal plates for large-sized objects such as vehicles. We have used a smaller-sized circular metal plate than for cases 3 and 4 as the illustration shows in Figure 7. The radius (*r*) of the plate equals 25 cm for case 5 and case 6, 35 cm for case 7 and case 8, 45 cm for case 9 and case 10, and 80 cm for case 11 and case 12. The magnetic field intensity along the measure line by the two WPT systems is present in Figure 7. We can conclude from the curves that the metal shield effect increases with the metal size by and large, yet, as the metal plate radius *r* ≤ 35 cm, the magnetic field intensity of the two WPT systems working anti-phase (case 6 and case 8) is stronger than in situations with no shield (case 2). The curves of cases 3, 4, 11, and 12 in Figure 7 indicate that large metal plates show an excellent shielding effect. Whether the metal plate shield will work or not is related to the coupling condition of the WPT coils as the plate size is approximate to the coils [34]. To guarantee the shield effect, the radius of the metal should be larger than 35 cm.

In conclusion, the magnetic field leakage in the middle region of the two WPT coils working anti-phase is stronger than that in-phase, but in the peripheral regions, the anti-phase leakage is weaker. Thin metallic plates of a large size compared to the WPT coils can shield the leakage of magnetic field well, while a small sized one may worsen the situation.

### 3.2. Induced Electric Field in the Human Body

**Figure 8:**
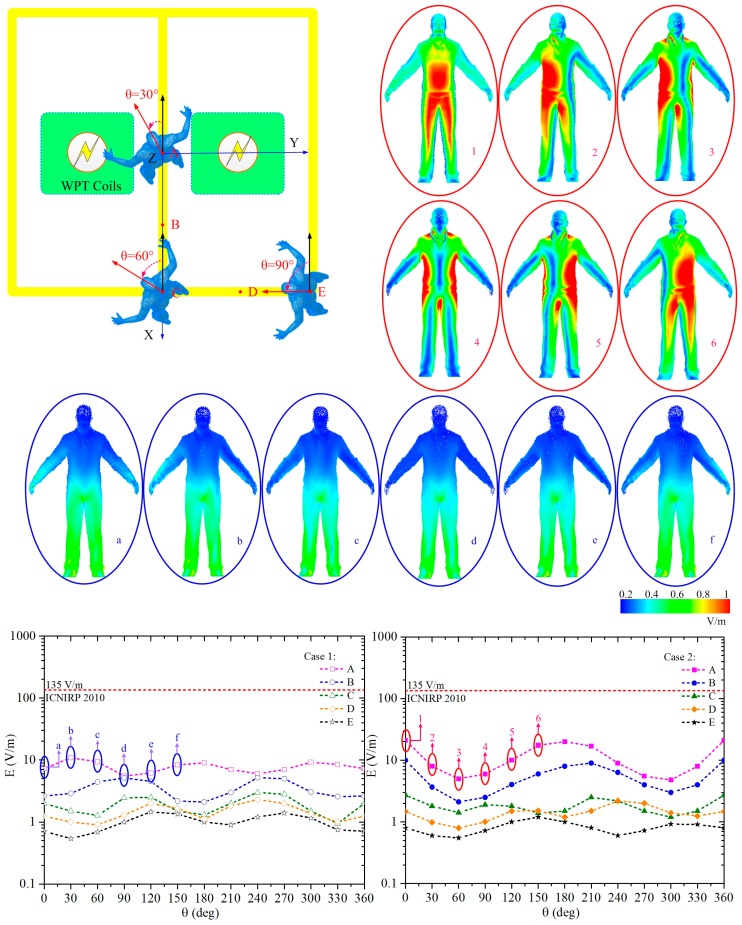
The maximum induced electric field in a standing human body with different orientations around the two WPT systems.

To restrict adverse effects to nerve cells and networks, the induced electric field is evaluated. The maximum induced electric fields in a standing human body at different orientations around the two WPT systems are shown in Figure 8. The orientation of the human body is varied in steps of 30°. An orientation (θ) of 0° corresponds to a human body facing towards the negative X axis. The human body stands at position A–E, and the exposure scenarios are cases 1 and 2. As Figure 8 shows, the maximum electric field appears when the human standing with θ equals 0° at position A, which is the middle of the two WPT systems working anti-phase as case 2 represents. The maximum magnitude of the electric field is 21.0 V/m, which is smaller than the basic restriction of 135 V/m by ICNIRP 2010. Figure 8 (a–f) shows the distribution of electric field induced in human body with different orientations standing at position A in case 1, and 1–6 for case 2. According to the curves and illustrations in Figure 8, we can see that the orientations will influence not only the maximum magnitude of induced electric field but also its distribution in human body.

In position A, the induced electric field is strong in trunk, groin and genitals of the human body in case 2 while it focuses on lower limbs in case 1. The distribution changes distinctly with the orientations in case 2 while it is almost unaltered in case 1. In the human head and trunk, there exist important tissues such as the central nervous system and organs, which are of greater concern compared to the limbs. Hence, it is safer for human standing at position A in case 1, in addition, it is more convenient to estimate and shield due to the constant distribution of different orientations.

**Figure 9:**
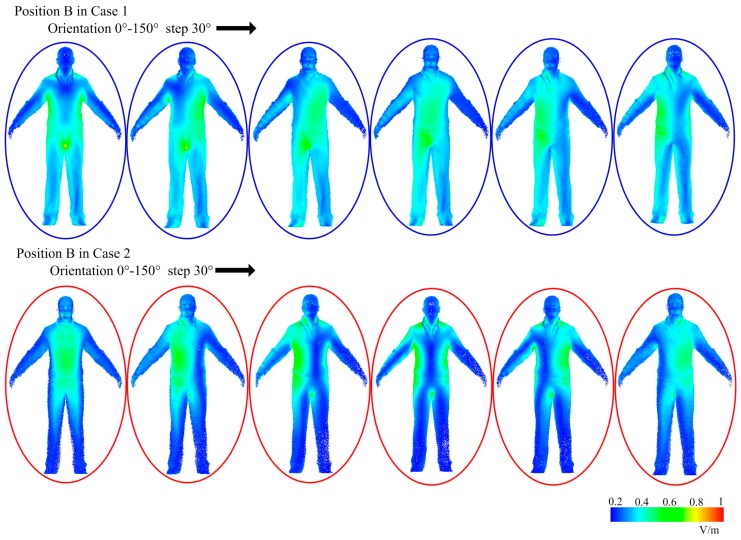
The induced electric field in a standing human body with different orientations at position B.

In position B, the induced electric field in human body is shown in Figure 9. For both case 1 and case 2, the electric field induced in trunk is relatively stronger than other body parts. The electric field induced in lower limbs in case 1 is stronger than in case 2. On the whole, when a human is standing at position B, it is safer to use the two WPT systems working anti-phase, as case 2 represents.

**Figure 10:**
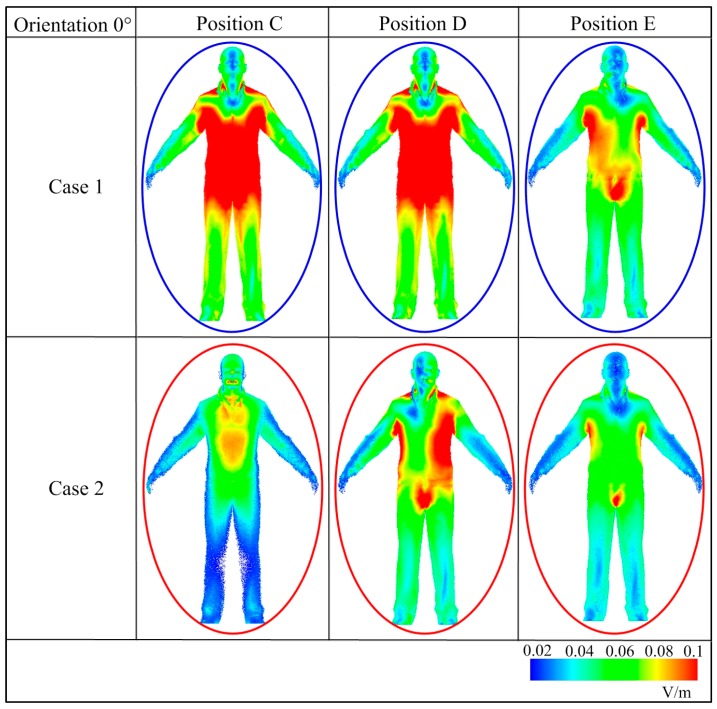
The induced electric field in a standing human body at positions C, D, and E.

The electric field induced in the human body standing at positions C, D and E is reasonably weak, as the curves show in Figure 8. The induced field in trunk, groin and genitals are also relatively stronger compared with other parts of human body, as Figure 10 shows. Taking a holistic look of the induced electric field in the whole human body, the mean value in case 1 is stronger than in case 2. Only an orientation of 0° is mapped while all others conform to the regularities of distribution, which are not shown in this paper.

In conclusion, as the human body is exposed to the two parallel WPT systems, the orientations of the human body will influence the maximum magnitude of induced electric field and its distribution in human body. The induced electric field centralizes in the trunk, groin, and genitals with only one exception: for the human body standing right at the middle of the two WPT coils working in-phase, the induced electric field focuses on lower limbs. The electric field induced in human body by the two WPT systems working in-phase is weaker than in anti-phase for the human body standing in the middle of the systems, but the result is not always tenable as the human moving around the WPT systems.

### 3.3. SAR in Typical Tissues

SAR is an important factor to be evaluated for preventing issue heating. ICNIRP 1998 exposure limits on SAR (W/kg) are time-averaged over any 6-min period: 0.08 for whole body, 2 for head and trunk, and 4 for limbs. The whole body average SAR shall be calculated as the ratio of the power dissipated in all tissues and fluids of the body divided by its total mass. In this paper, all presented SAR values in tissues are the peak spatial-average SAR [39,40,41] calculated for cases when the systems working continuously with stable charging power. The magnitudes of the complex electric field amplitudes are used as Equation (4) shows; the actual time-averaged values of the SAR are lower than those presented. The peak spatial-average SAR values in typical tissues of human body standing at different positions with different orientations in case 1 and case 2 are listed in Table 4, Table 5, Table 6, Table 7 and Table 8. The values are far below ICNIRP 1998 limits. By comparing the average values in Table 4, Table 5, Table 6, Table 7 and Table 8, we can conclude that the SAR in a human standing at position A in case 2 is much larger than that in case 1, but it does not appear to be much different at other positions. The value in the lungs always seem to be larger than other organs, while the value in the liver is minimal. According to previous studies, the spatial peak SAR values exceed a whole-body average value by a factor greater than 20 [41], the whole-body average value will also satisfy the guidelines constraint which is ignored in this paper. In addition, to obtain the SAR values for a different or the maximum accept-power level, the SAR results can be adjusted by scaling the results [39] using Equation (6).

(6) $$SAR_{d}=SAR_{c}(P_{d}/P_{c})$$

where *P_d_* is the desired accepted power, *P_c_* is the computed power, *SAR_d_* is the scaled SAR, and *SAR_c_* is the computed SAR.

Taking *SAR_d_* 2 W/kg, *SAR_c_* 0.119 W/kg from Table 4, *P_c_* 3.3 kW, we can calculate that the maximum accept-power is 55.5 kW. In consideration of the maximum magnitude of the induced electric field 21.0 V/m from Figure 8 and the basic restriction 135 V/m, the corresponding maximal allowable power is 136.4 kW. The unperturbed leakage of the magnetic field is closely related to the position depending on the study in Section 3.1. As the reference level is too conservative in ICNIRP 1998, the maximum accepted power is 3.1 kW considering the peak magnetic field 0.75 A/m from Figure 4 and the reference level 0.73 A/m. Since the magnetic field in the frequency range of 100 kHz to 10 MHz does not contribute significantly to the possibility of shocks, burns, or surface charge effects, the field strength values can be exceeded provided that the basic restrictions are met and adverse indirect effects can be excluded, hence, the induced electric field and SAR are foremost concerned from the perspective of protecting human exposure to the WPT systems while the distribution of the unperturbed leakage of the magnetic field provides a forecast of probably threatening regions. From the above, as the human body standing around the charging stations from positions A–E, the charging power should be no larger than 55.5 kW.

## 4. Conclusions

In order to ensure that the parallel WPT systems are safely approachable, it is critical that the systems comply with the relevant regulations. In this study, the magnetic field leakage from parallel WPT systems working with phase difference is predicted, and its effects on the human body (such as induced electric field and SAR) are also evaluated. The results show that the magnetic field is weaker in the middle region between the two WPT systems working in-phase and in the peripheral regions around the WPT systems working anti-phase. Thin metallic plates of large size compared to the WPT coils can shield the leakage of the magnetic field well, while small sized ones may worsen the situation. The orientations of the human body will influence the maximum magnitude of the induced electric field and its distribution in human body. The induced electric field centralizes in the trunk, groin, and genitals with only one exception: when the human body stands right in the middle of the two WPT coils working in-phase, the induced electric field focuses on the lower limbs. The SAR value in the lungs always seems to be greater than in other organs, while the value in the liver is minimal. The maximum accept-power is 55.5 kW to meet the basic restrictions of ICNIRP guidelines and 3.1 kW to meet the reference levels. The reference level is too conservative and can be exceeded provided that the basic restrictions are met and adverse indirect effects can be excluded. The conclusions in this paper will be useful for security considerations with respect to parallel WPT systems working simultaneously.

## Figures and Tables

**Table 1:** Coil parameters without considering the metal effects.

*f* = 1 MHz	Self-Inductance (μH)	Resistance (mΩ)	Matched Capacitance (nF)	Coupling Coefficient
Tx loop	1.196	12.316	21.170	*k*_12_ = 0.608 *k*_23_ = 0.173 *k*_34_ = 0.607
Tx coil	21.938	106.660	1.155	
Rx coil	21.938	104.233	1.155	
Rx loop	1.197	12.126	21.154	

**Table 2:** Parameters of typical tissues at the operating frequency (1 MHz).

Tissues of Human Model	Relative Permittivity	Relative Permeability	Conductivity (S/m)	Density (kg/m^3^)
Fat	50.8	1	0.044	911
Muscle	1840	1	0.503	1090
Brain	860	1	0.163	1045
Heart	1970	1	0.328	1081
Liver	1540	1	0.187	1079
spleen	2290	1	0.182	1089
Lung	733	1	0.136	394
Kidney	2250	1	0.278	1066
Bone	145	1	0.024	1908

**Table 3:** Currents in WPT coils. RMS: root mean square.

*V*_1_ = 925.5 V *P_o_* = 3.3 kW	RMS Value (A)	Phase (Degree)
*I*_1_	13.5	−4.4
*I*_2_	13.9	−69.8
*I*_3_	9.7	−169.4
*I*_4_	18.2	118.1

**Table 4:** The peak spatial-average specific absorption rate (SAR) values (W/kg) in typical tissues with different orientations in position A.

Position A Case 1 Case 2	0°	60°	120°	180°	240°	300°	Average
Brain	4.5 × 10^−7^ 1.6 × 10^−5^	6.8 × 10^−7^ 2.8 × 10^−5^	6.2 × 10^−7^ 2.1 × 10^−5^	4.0 × 10^−7^ 1.5 × 10^−5^	3.0 × 10^−7^ 1.6 × 10^−5^	2.9 × 10^−7^ 2.1 × 10^−5^	4.6 × 10^−7^ 2.0 × 10^−5^
Heart	3.2 × 10^−7^ 4.9 × 10^−5^	3.6 × 10^−7^ 5.6 × 10^−5^	4.5 × 10^−7^ 4.5 × 10^−5^	1.9 × 10^−7^ 4.4 × 10^−5^	1.6 × 10^−7^ 4.7 × 10^−5^	4.9 × 10^−7^ 4.9 × 10^−5^	3.3 × 10^−7^ 4.8 × 10^−5^
Liver	2.1 × 10^−10^ 1.7 × 10^−8^	4.0 × 10^−10^ 2.1 × 10^−8^	1.8 × 10^−10^ 2.5 × 10^−8^	2.1 × 10^−10^ 1.7 × 10^−8^	4.0 × 10^−10^ 2.3 × 10^−8^	1.9 × 10^−10^ 2.6 × 10^−8^	2.7 × 10^−10^ 2.2 × 10^−8^
spleen	7.3 × 10^−7^ 1.1 × 10^−5^	7.0 × 10^−7^ 6.9 × 10^−5^	5.8 × 10^−7^ 1.2 × 10^−4^	8.8 × 10^−7^ 9.9 × 10^−6^	6.7 × 10^−7^ 7.2 × 10^−5^	7.1 × 10^−7^ 1.1 × 10^−4^	7.1 × 10^−7^ 6.5 × 10^−5^
Lung	2.5 × 10^−6^ 1.6 × 10^−4^	3.5 × 10^−6^ 3.9 × 10^−4^	2.2 × 10^−6^ 3.2 × 10^−4^	2.9 × 10^−6^ 1.6 × 10^−4^	3.5 × 10^−6^ 4.5 × 10^−4^	3.0 × 10^−6^ 3.2 × 10^−4^	2.9 × 10^−6^ 3.0 × 10^−4^
Kidney	1.0 × 10^−6^ 3.2 × 10^−5^	8.0 × 10^−7^ 8.6 × 10^−5^	7.7 × 10^−7^ 1.2 × 10^−4^	1.0 × 10^−6^ 3.3 × 10^−5^	1.1 × 10^−6^ 8.8 × 10^−5^	9.3 × 10^−7^ 1.1 × 10^−4^	9.3 × 10^−7^ 7.8 × 10^−5^
Head, trunk and limbs	0.014 0.194	0.020 0.016	0.009 0.282	0.005 0.092	0.020 0.028	0.019 0.104	0.015 0.119

**Table 5:** The peak spatial-average SAR values (W/kg) in typical tissues with different orientations in position B.

Position B Case 1 Case 2	0°	60°	120°	180°	240°	300°	Average
Brain	4.3 × 10^−6^ 2.5 × 10^−6^	5.0 × 10^−6^ 3.0 × 10^−6^	4.2 × 10^−6^ 2.6 × 10^−6^	3.7 × 10^−6^ 4.2 × 10^−6^	4.0 × 10^−6^ 2.1 × 10^−6^	6.2 × 10^−6^ 2.7 × 10^−6^	4.6 × 10^−6^ 2.9 × 10^−6^
Heart	3.9 × 10^−6^ 8.2 × 10^−6^	7.9 × 10^−6^ 5.0 × 10^−6^	7.3 × 10^−6^ 1.1 × 10^−5^	4.3 × 10^−6^ 6.8 × 10^−6^	7.6 × 10^−6^ 6.7 × 10^−6^	1.9 × 10^−5^ 6.9 × 10^−6^	8.3 × 10^−6^ 7.4 × 10^−6^
Liver	3.0 × 10^−9^ 3.6 × 10^−9^	3.7 × 10^−9^ 3.5 × 10^−9^	3.9 × 10^−9^ 1.8 × 10^−9^	2.5 × 10^−9^ 3.8 × 10^−9^	3.4 × 10^−9^ 4.8 × 10^−9^	3.8 × 10^−9^ 1.5 × 10^−9^	3.4 × 10^−9^ 3.2 × 10^−9^
spleen	1.4 × 10^−6^ 1.2 × 10^−5^	1.1 × 10^−5^ 5.4 × 10^−6^	1.4 × 10^−5^ 4.6 × 10^−6^	1.5 × 10^−6^ 8.9 × 10^−6^	1.4 × 10^−5^ 5.7 × 10^−6^	1.4 × 10^−5^ 5.1 × 10^−6^	9.3 × 10^−6^ 7.0 × 10^−6^
Lung	2.5 × 10^−5^ 6.5 × 10^−5^	4.8 × 10^−5^ 2.3 × 10^−5^	5.1 × 10^−5^ 3.6 × 10^−5^	1.9 × 10^−5^ 3.5 × 10^−5^	6.2 × 10^−5^ 2.3 × 10^−5^	5.2 × 10^−5^ 2.4 × 10^−5^	4.3 × 10^−5^ 3.4 × 10^−5^
Kidney	4.2 × 10^−6^ 1.4 × 10^−5^	1.0 × 10^−5^ 8.6 × 10^−6^	2.2 × 10^−5^ 6.2 × 10^−6^	4.7 × 10^−6^ 7.4 × 10^−6^	1.5 × 10^−5^ 8.4 × 10^−6^	1.8 × 10^−5^ 7.8 × 10^−6^	1.2 × 10^−5^ 8.7 × 10^−6^
Head, trunk and limbs	0.085 0.002	0.006 0.012	0.020 0.056	0.018 0.001	0.010 0.008	0.007 0.015	0.024 0.016

**Table 6:** The peak spatial-average SAR values (W/kg) in typical tissues with different orientations in position C.

Position C Case 1 Case 2	0°	60°	120°	180°	240°	300°	Average
Brain	5.2 × 10^−7^ 3.4 × 10^−7^	5.8 × 10^−7^ 4.7 × 10^−7^	5.5 × 10^−7^ 4.1 × 10^−7^	9.0 × 10^−7^ 2.7 × 10^−7^	5.7 × 10^−7^ 3.9 × 10^−7^	5.6 × 10^−7^ 5.4 × 10^−7^	6.1 × 10^−7^ 4.0 × 10^−7^
Heart	4.2 × 10^−7^ 5.0 × 10^−7^	1.2 × 10^−6^ 4.0 × 10^−7^	1.2 × 10^−6^ 4.4 × 10^−7^	8.6 × 10^−7^ 3.9 × 10^−7^	1.4 × 10^−6^ 7.7 × 10^−7^	8.7 × 10^−7^ 3.5 × 10^−7^	9.9 × 10^−7^ 4.8 × 10^−7^
Liver	3.8 × 10^−10^ 1.3 × 10^−10^	3.7 × 10^−10^ 1.9 × 10^−10^	4.1 × 10^−10^ 2.1 × 10^−10^	6.5 × 10^−10^ 1.1 × 10^−10^	3.9 × 10^−10^ 2.2 × 10^−10^	9.0 × 10^−10^ 1.9 × 10^−10^	5.2 × 10^−10^ 1.8 × 10^−10^
spleen	1.5 × 10^−6^ 5.9 × 10^−8^	7.8 × 10^−7^ 5.5 × 10^−7^	7.2 × 10^−7^ 4.9 × 10^−7^	1.6 × 10^−6^ 6.7 × 10^−8^	7.5 × 10^−7^ 5.4 × 10^−7^	9.5 × 10^−7^ 5.6 × 10^−7^	1.1 × 10^−6^ 3.2 × 10^−7^
Lung	1.2 × 10^−5^ 9.0 × 10^−7^	4.9 × 10^−6^ 2.4 × 10^−6^	3.4 × 10^−6^ 4.0 × 10^−6^	5.4 × 10^−6^ 1.6 × 10^−6^	3.9 × 10^−6^ 3.2 × 10^−6^	4.8 × 10^−6^ 3.0 × 10^−6^	5.7 × 10^−6^ 2.5 × 10^−6^
Kidney	1.5 × 10^−6^ 1.8 × 10^−7^	1.5 × 10^−6^ 4.9 × 10^−7^	7.6 × 10^−7^ 1.0 × 10^−6^	1.1 × 10^−6^ 2.1 × 10^−7^	8.0 × 10^−7^ 6.2 × 10^−7^	1.2 × 10^−6^ 5.2 × 10^−7^	1.1 × 10^−6^ 5.0 × 10^−7^
Head, trunk and limbs	1.5 × 10^−4^ 1.0 × 10^−2^	2.3 × 10^−3^ 5.0 × 10^−4^	4.6 × 10^−3^ 9.4 × 10^−4^	2.2 × 10^−4^ 2.3 × 10^−3^	5.1 × 10^−3^ 1.2 × 10^−3^	1.6e-2 6.4 × 10^−3^	4.7 × 10^−3^ 2.6 × 10^−3^

**Table 7:** The peak spatial-average SAR values (W/kg) in typical tissues with different orientations in position D.

Position D Case 1 Case 2	0°	60°	120°	180°	240°	300°	Average
Brain	6.1 × 10^−7^ 5.2 × 10^−7^	8.4 × 10^−7^ 4.6 × 10^−7^	6.6 × 10^−7^ 4.6 × 10^−7^	6.4 × 10^−7^ 4.6 × 10^−7^	6.4 × 10^−7^ 2.8 × 10^−7^	6.7 × 10^−7^ 4.1 × 10^−7^	6.8 × 10^−7^ 4.3 × 10^−7^
Heart	4.4 × 10^−7^ 5.2 × 10^−7^	1.2 × 10^−6^ 6.8 × 10^−7^	1.2 × 10^−6^ 3.9 × 10^−7^	1.3 × 10^−6^ 6.5 × 10^−7^	1.2 × 10^−6^ 5.6 × 10^−7^	1.1 × 10^−6^ 7.2 × 10^−7^	1.1 × 10^−6^ 5.9 × 10^−7^
Liver	3.7 × 10^−10^ 2.7 × 10^−10^	4.0 × 10^−10^ 2.1 × 10^−10^	2.8 × 10^−10^ 2.4 × 10^−10^	5.7 × 10^−10^ 3.1 × 10^−10^	3.6 × 10^−10^ 1.8 × 10^−10^	2.8 × 10^−10^ 2.2 × 10^−10^	3.8 × 10^−10^ 2.4 × 10^−10^
spleen	1.6 × 10^−6^ 8.0 × 10^−7^	6.6 × 10^−7^ 1.3 × 10^−7^	6.5 × 10^−7^ 1.0 × 10^−6^	1.6 × 10^−6^ 9.8 × 10^−7^	7.8 × 10^−7^ 2.0 × 10^−7^	9.5 × 10^−7^ 9.1 × 10^−7^	1.0 × 10^−6^ 6.7 × 10^−7^
Lung	1.1 × 10^−5^ 4.4 × 10^−6^	4.2 × 10^−6^ 1.8 × 10^−6^	3.9 × 10^−6^ 3.2 × 10^−6^	5.7 × 10^−6^ 3.2 × 10^−6^	3.2 × 10^−6^ 1.6 × 10^−6^	6.0 × 10^−6^ 5.7 × 10^−6^	5.7 × 10^−6^ 3.3 × 10^−6^
Kidney	2.1 × 10^−6^ 1.4 × 10^−6^	1.7 × 10^−6^ 5.2 × 10^−7^	8.7 × 10^−7^ 6.2 × 10^−7^	1.1 × 10^−6^ 8.1 × 10^−7^	9.5 × 10^−7^ 4.0 × 10^−7^	1.4 × 10^−6^ 9.7 × 10^−7^	1.4 × 10^−6^ 7.9 × 10^−7^
Head, trunk and limbs	6.0 × 10^−4^ 1.1 × 10^−3^	2.3 × 10^−3^ 1.7 × 10^−3^	5.9 × 10^−3^ 1.4 × 10^−3^	3.6 × 10^−4^ 4.6 × 10^−4^	1.2 × 10^−3^ 1.3 × 10^−3^	2.2 × 10^−3^ 6.9 × 10^−4^	2.1 × 10^−3^ 1.1 × 10^−3^

**Table 8:** The peak spatial-average SAR values (W/kg) in typical tissues with different orientations in position E.

Position E Case 1 Case 2	0°	60°	120°	180°	240°	300°	Average
Brain	2.3 × 10^−7^ 1.4 × 10^−7^	2.7 × 10^−7^ 1.6 × 10^−7^	1.8 × 10^−7^ 1.3 × 10^−7^	3.8 × 10^−7^ 2.3 × 10^−7^	3.2 × 10^−7^ 1.9 × 10^−7^	2.3 × 10^−7^ 1.5 × 10^−7^	2.7 × 10^−7^ 1.7 × 10^−7^
Heart	2.0 × 10^−7^ 1.1 × 10^−7^	5.1 × 10^−7^ 3.9 × 10^−7^	4.7 × 10^−7^ 2.6 × 10^−7^	4.1 × 10^−7^ 2.5 × 10^−7^	4.7 × 10^−7^ 2.6 × 10^−7^	2.9 × 10^−7^ 2.3 × 10^−7^	3.9 × 10^−7^ 2.5 × 10^−7^
Liver	1.3 × 10^−10^ 9.2 × 10^−11^	3.3 × 10^−10^ 9.7 × 10^−11^	2.3 × 10^−10^ 8.2 × 10^−11^	1.8 × 10^−10^ 1.3 × 10^−10^	1.5 × 10^−10^ 9.1 × 10^−11^	3.3 × 10^−10^ 2.4 × 10^−10^	2.1 × 10^−10^ 1.2 × 10^−10^
spleen	4.7 × 10^−7^ 3.0 × 10^−7^	3.5 × 10^−7^ 1.9 × 10^−7^	2.0 × 10^−7^ 1.5 × 10^−7^	4.6 × 10^−7^ 3.1 × 10^−7^	3.2 × 10^−7^ 1.8 × 10^−7^	2.6 × 10^−7^ 1.9 × 10^−7^	3.4 × 10^−7^ 2.2 × 10^−7^
Lung	3.5 × 10^−6^ 2.3 × 10^−6^	2.2 × 10^−6^ 1.1 × 10^−6^	1.1 × 10^−6^ 7.9 × 10^−7^	1.7 × 10^−6^ 1.2 × 10^−6^	1.5 × 10^−6^ 1.0 × 10^−6^	1.3 × 10^−6^ 9.9 × 10^−7^	1.9 × 10^−6^ 1.2 × 10^−6^
Kidney	5.4 × 10^−7^ 3.2 × 10^−7^	6.8 × 10^−7^ 4.2 × 10^−7^	3.1 × 10^−7^ 1.7 × 10^−7^	4.3 × 10^−7^ 2.6 × 10^−7^	6.4 × 10^−7^ 3.0 × 10^−7^	4.1 × 10^−7^ 2.8 × 10^−7^	5.0 × 10^−7^ 2.9 × 10^−7^
Head, trunk and limbs	1.2 × 10^−4^ 8.3 × 10^−5^	3.2 × 10^−4^ 3.0 × 10^−4^	1.8 × 10^−3^ 1.2 × 10^−3^	3.4 × 10^−2^ 6.2 × 10^−3^	2.8 × 10^−3^ 2.8 × 10^−3^	5.1 × 10^−3^ 3.0 × 10^−3^	7.4 × 10^−3^ 2.3 × 10^−3^
